# Initial development and validation of item banks to measure problematic hypersexuality

**DOI:** 10.12688/openreseurope.16131.3

**Published:** 2024-11-22

**Authors:** Piet van Tuijl, Peter Verboon, Jacques van Lankveld

**Affiliations:** 1 Psychology, Open University of The Netherlands, Heerlen, Limburg, The Netherlands

**Keywords:** Problematic hypersexuality; Non-problematic hypersexuality; Item Response Theory; Emotion dysregulation; Diagnostic accuracy.

## Abstract

**Background:**

Problematic Hypersexuality (PH) is defined as a distress caused by hypersexuality, to the extent that seeking treatment is considered. PH was previously measured with instruments stemming from different perspectives on problems related to hypersexuality. These instruments might best be analyzed in unison to discover the most optimal set of characteristics to measure PH.

**Methods:**

A total of 58 items were investigated with Item Response Theory (IRT). We included 1211 participants (592 women, 618 men, 1 other) from a representative Dutch general population sample of 18 years or older. In addition, 371 participants (116 women, 253 men, 2 other) in a web-based survey who sought information on their current level of PH were included. This latter group was divided into those that did or did not consider treatment and group differences in item averages were assessed.

**Results:**

After item selection, 26 out of 58 items were retained and divided in two scales: Emotion Dysregulation-PH – 9 items representing the distressing emotional patterns coinciding with hypersexual preoccupation – and Negative Effects-PH – 17 items representing the negative consequences of patterns of hypersexual thoughts and behavior. Assumptions for IRT analyses were met (unidimensionality, local independence and monotonicity). After an IRT graded response model was fit, the scales showed sufficient reliability for the target population of hypersexual individuals. In the general population the scales showed large floor effects and were less reliable.

**Conclusions:**

With this study a first step is taken in validating two complementary item banks to measure PH. Further development of the item banks should include the investigation of responsiveness. New items should be constructed to assess less-explored areas of PH and improve differentiating power of the scales. This study showed that diagnostic accuracy for PH is currently difficult to attain with a survey, even when using an extended item set representing the most unique characteristics of PH.

## Plain language summary

A survey to investigate hypersexuality was developed. The survey intends to distinguish between those experiencing problems due to hypersexuality (Problematic Hypersexuality; PH) and others who are also hypersexual but don't experience problems (Non-problematic Hypersexuality; NH). We investigated 58 items for final inclusion in the survey, of which 26 adequately distinguished between PH and NH. These 26 items were divided in two scales: Emotion Dysregulation-PH, measuring the negative emotional patterns that can occur in hypersexuality, and Negative Effects-PH, measuring the negative consequences that can occur in hypersexuality. Psychometric characteristics of each item were investigated and all items showed sufficient validity to be included in the scales. The survey should specifically be used to assess the level of problems hypersexual individuals experience, and is of less use in the general population. In future studies it needs to be investigated if progress in therapy can be measured with this instrument. Our study showed that even when the most optimal set of items is used to measure problems due to hypersexuality, it still is hard to distinguish between hypersexual individuals experiencing problems and those not experiencing problems due to hypersexuality. The survey can offer a quick descriptive assessment of the level of problematic hypersexuality that is experienced and thus can efficiently aid health care providers working with people experiencing problems due to hypersexuality.

## Introduction

Problematic Hypersexuality (PH) has been preliminarily defined as the experience of distress and negative consequences due to hypersexual urges and behavior - to the extent that it causes the individual to at least consider seeking help ( Van Tuijl *et al*., 2023a). An extended and testable definition can be found here. This definition describes PH as consisting of ten trait characteristics that originate in and are maintained by two driving forces of PH: high sexual desire and emotion dysregulation ( Kingston, 2018). The ten characteristics are based on three diverging diagnostic perspectives on PH: sex addiction ( Goodman, 1993), Hypersexual Disorder (HD; Kafka, 2010) and Compulsive Sexual Behavior Disorder (CSBD; Kraus *et al*., 2018). Of these three, only the diagnosis of CSBD has been officially included in a classification system – the ICD-11 ( WHO, 2019). This seems to suggest that criteria unique to the other two diagnoses are invalid to differentiate PH from other conditions. However, this has not been tested in samples appropriate to investigate the differentiating power of these criteria. In the current study we investigated a web-based sample that consisted of a relatively large number of participants who were afflicted by PH. Also included in this sample were Non-problematic Hypersexual (NH) participants: those experiencing high levels of sexual desire, but without the high levels of related distress ( Briken *et al.*, 2022; Spenhoff *et al*., 2013; Štulhofer *et al*., 2016; van Zessen, 1995). We have added a large representative sample from the Dutch population of 18 years and older to investigate hypersexuality in the general population as well. We adhered to Patient Routine Outcome Measurement Information System (PROMIS, Cella *et al.*, 2007) validation guidelines ( Prinsen *et al*., 2018) using Item Response Theory (IRT) techniques. We targeted an ideally aligning twofold aim in this study: 1) psychometrically test items that measure problems due to hypersexuality – thus developing and validating item banks for PH; and 2) finetune the preliminary definition of PH, by taking into account previous conceptualizations – Sex Addiction, Hypersexual Disorder and Compulsive Sexual Behavior Disorder. The main research gap this study addresses concerns the lack of integration of different perspectives on PH. Empirical studies into the combined indicators of PH are lacking – hypersexuality studies mainly focused on one of the three perspectives mentioned above. The current study puts to practice what has been previously suggested ( Montgomery-Graham, 2017) and combines different questionnaires previously developed for one of the three perspectives on PH. This combination allows us to test each questionnaires’ items in one study, not to pit the three perspectives against one another, but to converge – after extensive data analysis – toward an overarching concept that contains both the overlapping and unique indicators of each of the perspectives. Relating to the research gap mentioned above, there are also two methodological strategies we apply that have not been applied before to validate item banks for hypersexuality questionnaires ( van Tuijl *et al*., 2023a): 1) our study is the first to use IRT to develop and validate item banks to measure PH or related constructs; 2) we make use of the distinction between PH and Non-problematic Hypersexuality (NH; Štulhofer *et al*., 2016; van Zessen, 1995) to select the best items. Concerning IRT, we mention that the use of this technique allows for a continuous back-and-forth between data and construct as new items can be added anytime to the item banks. Concerning the distinction between PH and NH, this distinction allows us to select items that have the most discriminating power to discern these relevant groups from each other. Previous hypersexuality validation research did not use such group comparisons and thus could not unequivocally be expected to discern PH from NH. Both the use of IRT and the distinction between PH and NH will allow us to better address the main research gap – lack of integration of different perspectives on PH. A number of challenges ensue when developing an item bank for PH: 1) There is a low base rate of PH in the general population (GP); 2) The factor structure of PH is not well determined; and 3) Previously developed instruments to measure PH – used in this study to start up item bank development – might be lacking in content validity. By addressing these three challenges we can initiate the development of an item bank for PH that is open to adaptations based on empirical input from future studies. In this way “[s]earching for clarity” ( Moser, 2013) with regard to “[s]exual addiction, sexual compulsivity, sexual impulsivity, or what?” ( Bancroft & Vukadinovic, 2004) can become a continuous instead of a contentious process. Previous diagnoses for PH have not led to an integral perspective on PH as sometimes criteria of emotion dysregulation were discarded ( Kraus *et al*., 2018), while in other cases criteria stemming from a sex addiction perspective were not included ( Kafka, 2010), without research confirming or disconfirming such choices. In the current study we investigate indicators from multiple perspectives ( Montgomery-Graham, 2017) on PH and use an appropriate sample to test the uniqueness of these indicators to PH.

The first challenge encountered when developing an item bank for PH is the low base rate of PH in the general population (GP). Percentages around 1 – 3 % have been reported in national representative samples and cohorts ( Rutgers, 2017; Skegg *et al*., 2010). This low base rate implicates difficulties with assessing cue-validity ( Rosch & Mervis, 1975), with cue-validity defined as the measure of uniqueness of a cue or characteristic to a certain category or subpopulation. Items developed using only a GP sample might be of little use to distinguish between PH and non-distressed subpopulations that closely resemble it. We argue that the target population for which a PH item bank should be developed ( De Vet *et al*., 2011) should reflect the context in which the instrument to measure PH will be used: the general practitioner’s or sexologist’s office where only those will come to seek information who doubt whether or not their sexual preoccupation should be considered problematic. A secondary aim of such a discriminatory instrument can be to assess prevalence in the general population as well, though caution is advised when applying general population measurement to individual cases, e.g. as a screener. In the current study we addressed difficulties due to the low base rate of PH by including relatively many participants who actively sought information regarding their level of PH, approximately half of whom also considered seeking help for it.

The second challenge encountered when developing an item bank for PH concerns its factor structure. In the current study we used previously developed instruments to start up item bank development and also added 14 items we developed ourselves. It is not clear what factor structure could be assumed for the combination of these instruments and new items. Therefore, following van Tuijl *et al*. (2020), we investigated if a number of items could be subsumed under a Negative Effects factor. In particular those items that addressed: 1) neglect of responsibilities; 2) negative consequences in life domains; 3) disregard for harm to self or others; 4) failure to stop the sexual behavior; 5) continue the sexual behavior despite negative consequences; 6) withdrawal symptoms; 7) continuation of sexual behavior despite loss of pleasure; and 8) tolerance. Furthermore, in the current study, previously and newly developed items representing emotion dysregulation in PH have been investigated. Among other aspects, these items address shame ( Adams & Robinson, 2001) and lack of self-esteem ( van Zessen, 2013) in PH as in particular these show diverging effects for PH and NH ( van Tuijl *et al*., 2022a). We also included items that reflect feeling stuck in behavior one cannot stop. If it proved to be that PH is best analyzed as a multi-factorial construct, we will perform validation research on each of the relevant factors separately.

The third challenge encountered when developing an item bank for PH concerns content validity of previously developed instruments that we will use to start up the item bank. Items need to reflect the intended construct as this is the most important property of an instrument ( Prinsen *et al*., 2018; Terwee *et al*., 2007). We present two examples of equivocal item content in the previously developed instruments that we will use in this study. We note that these items can lead to problems with cue-validity as they might not be able to properly distinguish between relevant subpopulations. In the first example we consider the Dissatisfaction scale of the CSBD-19, which purports to measure continuation of sexual behavior even though the person “derives little or no satisfaction from it” ( Kraus *et al*., 2018, p. 109). We note that the three items of this scale (e.g. “Although sex was not as satisfying for me as before, I engaged in it”) might be answered affirmatively by other groups besides PH – such as those managing sexual desire discrepancy in a romantic relationship ( Vowels & Mark, 2020). Given the base rate of the two groups – higher for sexual desire discrepancy than for PH – this might lead to confounding results. In the second example of problematic content validity, we consider the use of sex to cope with dysphoric feelings, as reflected in the Coping scale of the HBI-19. The items of this scale (e.g. “Doing something sexual helps me feel less lonely”) might rather measure “sex used as coping” – something extant in the general population as well ( Van Tuijl *et al*., 2022b) – instead of “sex used as coping in PH”. If this is indeed the case, items for the Coping subscale will lack cue-validity. In the current study we will use both statistical and content analyses to decide on adequate and cue-valid item content for PH.

The goal of this study was to start the development of an item bank to measure PH and to test the preliminary definition of PH by assessing what cue-valid characteristics set PH apart from other conditions. As a gold standard, we used the need for help for PH – as indicated by study participants. Referring to a gold standard allows us to assess the uniqueness of characteristics to PH. With this item bank development and validations study, we hope to streamline discussions with regard to different perspectives on PH, by introducing a methodology – exemplified in the current study – that allows for investigations of separate indicators for PH, illustrating how there can be a continuous back-and-forth between theory and data.

## Methods

### Ethics

Ethical approvement for this study has been provided by the Institutional Review Board of the Open University of the Netherlands (approval number: U202008692). Written informed consent was obtained from all participants.

### Participants and procedure

Between February 2022 and February 2023, visitors of a Dutch-based online platform for psychological help for PH completed a survey that provided them with feedback on their current level of PH. The survey, owned by the Nederlands Centrum Voor Seksverslaving (NCVS, Dutch Center for Sex Addiction), targeted those in doubt about their level of “addiction to sex” (“seksverslaving”). It is expected that also those preoccupied with sex in a non-distressing way will seek information through the survey, as mechanisms of stigmatizing high sexual frequency (e.g. “designated patient”, Cantor *et al*., 2013) might have propagated self-doubt. Only first-time responses of participants who provided informed consent and were 18 years or older were included. Of the original 441 completed surveys a total of 42 non-first-time responses were excluded as well as 28 responses of participants indicating to be younger than 18, leaving a total of n= 371 completed surveys. In December 2022, a second sample was collected consisting of panel-data representative of the general Dutch population of 18 and older. A total of n=1211 completed surveys were collected. Panel participants completed the same survey as used in the web-based sample. Part of the original panel sample of n=1211 completed the survey a second time after 2 to 3 weeks (n=106) and another part (n = 109) completed the survey a second time after 4 to 5 weeks. Only first-time responses of panel participants have been included in the current study. A total of n=1582 fully completed surveys were retained for further analysis. There are important differences in data collection between the two samples. In the general population sample the gender proportion was divided in women 48.9 %, men 51.0 %, other 0.1 %. In the web-based sample it was women 31.3 %, men 68.2 %, other 0.5 %. Furthermore, the percentages of Kinsey scale scores differed in both samples, with the general population sample showing more exclusively heterosexual participants than the web-based sample (82% versus 61%). There were no large differences in percentages of exclusively homosexual - 3% in the general population sample versus 4% in the web-based sample. In the supplemental material we have added a histogram of the spread of Kinsey scale scores in both samples and added more detailed information. While the general population sample can be taken to be representative of the Dutch population of 18 years and older, the web-based sample must be seen as a self-selected convenience sample. However, we can assume that all who have finished the questionnaire in the web-based sample, had a need for information and feedback pertaining to their problems due to hypersexuality.

### Development and validation of scales using IRT

In this study we use IRT to develop and validate items banks for PH, because this offers certain advantages over more common validation practices. IRT has previously been used to develop and validate scales for general health surveys questionnaires focused on constructs such as fatigue, pain, and depressive symptoms (Cella et al., 2007; Luijten et al., 2020) and similarly might be used to develop and validate an item bank for PH as well. In contrast to validation using only factor analysis, IRT allows for the ordering and selection of items based on item parameters (e.g. item difficulty or differentiating power). Because item difficulty and individuals’ latent trait scores are located on the same scale, it can be assessed which items are optimal to measure the trait in relevant target populations (e.g. Alons et al., 2022). Information gained from IRT can also be used to develop diverging short forms, serving discriminative or evaluative purposes (Terwee et al., 2007). No studies have been undertaken, to our knowledge, that use IRT to develop measurement instruments for PH or related constructs.

### Measures

*General description.* Three instruments – the Pathos ( Carnes *et al*., 2012), the Hypersexual Behavior Inventory ( Reid *et al*., 2011) and the Compulsive Sexual Behavior Disorder scale ( Böthe *et al*., 2020) – measuring different aspects of PH have been completed by all participants. Added to these three instruments were 14 items representing characteristics of PH that were not addressed in the three instruments. In total, 58 items assessing different aspects of PH were included. For all instruments, higher scores indicated higher levels of the construct (e.g. more problematic hypersexual behavior). Additionally, data on age, gender, relationship status and sexual orientation were collected. Gender assessment was based on self-report, using the item “I am a …woman/man/other”. Data were also collected on sexual frequency, sexual satisfaction, number of sexual partners, use of porn, specification of the type(s) of problematic hypersexual behavior, sexual excitation- and inhibition proneness, psychological symptoms, and the need for professional help due to PH. This information was used to describe the samples and to differentiate between the GP, NH and PH subgroups.

*Pathos ( Carnes et al., 2012).* The Pathos is a one-scale, 6-item instrument, which was developed as a short screener to measure sex addiction (example item: “Do you hide some of your sexual behavior from others?”). The original version offered dichotomous answer options. In order to align outcomes with the other questionnaires, we changed the original dichotomous answer options (“Yes” or “No”) into five-point Likert scales (from “Never” (= 1) to “Very often” (= 5)).

*Hypersexual Behavior Inventory (HBI-19; Reid et al., 2011).* The HBI-19 is a three-scale, 19-item instrument, which was developed to measure hypersexual behavior. Its three subscales are: Control (eight items, e.g. “My attempts to change my sexual behavior fail “), Coping (seven items, e.g. “I use sex to forget about the worries of daily life “) and Consequences (four items, e.g. “I engage in sexual activities that I know I will later regret “). Items are rated on five-point Likert scales (from “Never” (= 1) to “Very often” (= 5)).

*Compulsive Sexual Behavior Disorder scale (CSBD-19; Böthe et al., 2020).* The CSBD-19 is a five-scale, 19-item instrument, developed to measure compulsive sexual behavior disorder. Its five subscales are: Control (three items, e.g. “I could not control my sexual cravings and desires”), Salience (three items, “Sex has been the most important thing in my life”), Relapse (three items, “I was not successful in reducing the amount of sex I had”), Dissatisfaction (three items, “I had sex even when I did not enjoy it”) and Negative Consequences (seven items, “I did not accomplish important tasks because of my sexual behavior”. Level of agreement with items is rated on four-point Likert scales (from “Totally disagree” (= 1) to “Totally agree” (=4)). We followed the Beaton protocol in translating the Pathos, the HBI-19 and the CSBD-19 into Dutch ( Beaton *et al*., 2000).

*Problematic Hypersexuality Items (PHI).* This set of 14 newly developed items were included to fill gaps left by the Pathos, the HBI-19 and the CSBD-19 in measuring PH. The items concern emotion dysregulation (e.g. “I feel I’m stuck in my patterns of sexual behavior”), withdrawal (“When I stop with sex I feel nervous and restless”), tolerance (“I feel my desire for sex increases”) and social aspects (e.g. “Others tell me that it would be better if I would stop with certain sexual behaviors”). Items are rated on five-point Likert scales (from “Never” (= 1) to “Very often” (= 5)). 

*Symptom Questionnaire (SQ-48; Carlier et al., 2012).* The SQ-48 is a nine-scale, 48-item instrument measuring psychological symptoms, distress and vitality in the past week. It consists of nine subscales. Seven subscales measure psychological symptoms (depression, anxiety, somatization, social phobia, agoraphobia, aggression and cognitive problems) and two subscales are related to work (or education) and vitality. In this study we made use of the total SQ score – measuring general distress (7 subscales) – and the subscale measuring depressive symptoms – termed “Mood” – which consists of 6 items (e.g. “I considered my death or suicide”). Items are rated on five-point Likert scales (from “Never” (= 0) to “Very often” (= 4)). Internal consistency (McDonald’s ω, McDonald, 1999) of the Mood subscale in the current study was .90.

*Sexual Inhibition/Sexual Excitation Scales – Short Form (SIS/SES-SF; Carpenter *et al.*, 2011).* The SISSES/SF is a three-scale, 14-item instrument developed to measure sexual excitation and inhibition proneness. Its three subscales measure sexual excitation proneness - SES (ω = .90), sexual inhibition proneness due to performance failure – SIS1 (ω = .74), and sexual inhibition proneness due to performance consequences – SIS2 (ω = .72). Level of agreement with items is rated on four-point Likert scales (from “Strongly disagree” (=1) to “Strongly agree” (= 4)). In the current study the SIS/SES-SF is used for descriptive purposes.

*Distinction between non-problematic and problematic hypersexual (NH and PH) groups.* Two items addressing the need for help were included in the survey. These items addressed the current treatment status in relation to PH and current need for help for PH (e.g. “Do you need help for sex addiction? Not at all / Almost not at all / A little bit / Quite a bit / Very much). We used the term “sex addiction” as this is the most common colloquial term in the population, though we are aware of theoretical concerns with regard to this terminology. From the sample collected via Sekned, we categorized as PH those indicating they needed at least some help for sex addiction. Others – indicating they did not need help - were categorized as NH. We expect that only hypersexual respondents participated via the site, assuming that only those interested in feedback would complete the survey (which took on average 23 minutes).

### Statistical analyses

*Descriptive analyses.* descriptive statistics will be provided per subsample (GP, NH and PH) on socio-demographic variables, sexual feelings and behavior and psychological symptoms. Effect sizes of differences between these subsamples for these variables will be based on t-tests (Cohen’s *d*).

Descriptive and other analyses for this study have been performed in R ( R Core Team, 2022). Gender differences and differences in sexual orientation have been taken into account in this study if this supported its primary psychometric objective. They are reported for *differential item functioning* (DIF) for women and men and sexual orientation (see below) and for *interpretability* of results (see below).

*Item selection.* Item mean and standard deviation (SD) per group were used to select items that differentiate well between NH and PH. Items were selected if average item scores for NH were at least 0.5 x pooled SD lower than average item scores for PH. This rule of thumb we based on 0.5 x SD being considered as the smallest detectable change (SDC; De Vet *et al.*, 2011, p. 259). If content validity of an item was considered high, while the difference in item mean in NH and PH was slightly smaller than 0.5 x SD (e.g. 0.45 x SD), we selected the item as well. Differences between GP and the other groups were not used to decide on item selection. The 11 ^th^ item of the HBI-19 only differed in tense from the first item of the CSBD-19; of these two items, the item showing the best differentiating power was retained.

*Structural validity.* After item selection, Confirmatory Factor Analysis (CFA) was performed, using the R package “lavaan” ( Rosseel, 2012). We tested the factor structure suggested by previous research ( Van Tuijl *et al*., 2020) and categorized all selected items accordingly. We modelled the selected items - if needed - as a multi-factor model and its fit was considered sufficient with a Comparative Fit Index (CFI) > .95, a Tucker-Lewis Index (TLI) > .95, a Standardized Root Mean Square Residual (SRMR) < .10 and a Root Mean Square Error of Approximation (RMSEA) < .08 ( Schermelleh-Engel *et al*., 2003). After confirming the factor structure, we evaluated internal consistency for each factor separately by calculating the explained common variance (ECV), the hierarchical omega (sufficient if > .7) and the eigenvalue ratio of the first and second factor (sufficient if > 4:1) using bi-factor and parallel analysis with the “psych” package ( Revelle, 2023). We then checked IRT assumptions of unidimensionality, local independence and monotonicity for each subscale – if there was more than one. For unidimensionality we used the same fit measures as before to decide on good fit: CFI > .95, TLI > .95, SRMR < .10 and RSMEA < .08.

Local dependence of item pairs was assessed by checking the percentage of item pairs on a scale that showed residual correlations > .2 ( Reeve *et al*., 2007). Monotonicity, expressing that higher latent trait scores go together with an increased probability to score higher on the item, was tested using Mokken scaling with the package “mokken” ( Van der Ark, 2007). The assumption of monotonicity was met when individual item values H _i_ > .3 and scale-H > .5. After assumptions were met, IRT analyses were performed using the R package “mirt” ( Chalmers, 2012) and applying the Graded Response Model (GRM) to factor(s). Discrimination and threshold parameters were estimated for each item, representing the differentiating power of an item and the range of latent trait scores on which the item functions most optimally. For item fit we used a p-value < .001 for the S-X ^2^ statistic as an indication of item misfit as compared to expected response frequencies ( Kang & Chen, 2011). 

*Latent trait reliability.* Reliability of the measurements of participants can be different for different levels of the latent trait(s). We investigated the standard error of measurement (SEM) to assess the reliability of individual measurements. Reliability was considered sufficient with a SEM < .0316, representing a reliability > .90 ( Luijten *et al*., 2020). Reliability was regarded separately for the GP and the combination of NH and PH. If multiple factors were found, relevant for the target population of NH and PH participants, for each of their resulting scales reliability analyses were performed.

*Differential Item Functioning (DIF).* To assess differential item functioning (DIF) – equal latent trait levels leading to differences in manifest scores between subgroups – we performed analyses for three contrasting subgroups for all relevant scales, using the R package “lordif” ( Choi, 2016). Firstly, we checked DIF for those identifying as women or men. Only three participants indicated a non-specified other gender identity – these were excluded from this analysis. Secondly, Secondly, we checked DIF for sexual orientation: we dichotomized the outcome of the Kinsey scale into a group that indicated to be exclusively heterosexual (n = 1229) and those indicating to be not exclusively heterosexual (n = 353). Thirdly, we checked DIF for the NH and PH subgroups. And fourthly, we checked DIF for the GP group versus the combination of the NH and PH group. Although the latter contrast is less relevant in establishing the functioning of scale(s) in the target population, it does allow us to assess how well the scale(s) functions in the GP, and this can serve as general population calibration of the item banks.

*Interpretability.* Scores per subsample (GP, NH and PH) per gender were calculated to allow for interpretability of scale(s) scores: qualitative meaning that can be given to quantitative outcomes ( Terwee *et al*., 2007). For the scale(s) also the Area Under the Curve (AUC) value was calculated for the NH – PH contrast. The AUC gives a measure of the ability of scales to distinguish between designated subpopulations.

*Construct validity.* Although item bank development for PH is in its initial stage, we do adhere to PROMIS guidelines ( Prinsen *et al*., 2018; Terwee *et al*., 2007) to test hypotheses regarding associations between scores of newly developed scale(s) and other relevant measures. We present four hypotheses to test the validity of the scale(s) that were constructed: 1) Those considering help for PH in the GP sample will show higher scores on the newly developed scale(s) than the rest of the GP sample. 2) Those considering help for PH in the GP sample will show higher scores on the newly developed scale(s) than participants from the GP sample who indicated they had 1 or more orgasms per day in the last 6 months. The cutoff of 1 or more orgasms per day can be seen as a proxy of hypersexual preoccupation ( Kafka, 1997). Given that this group does not consider seeking help, their hypersexual interest can be considered non-problematic. Participants who considered help and have had 1 or more orgasms per day were included in the PH group. 3) The new scale(s) scores will show a weak to moderate (GP) or a moderate to strong (NH and PH) correlation with the Symptom Questionnaire – Mood subscale (depressive symptoms). 4) In linear regression analysis we expect a significant interaction between orgasm frequency and subpopulation (GP, NH and PH) in a regression model(s) with the new scale(s) as outcome. The last hypothesis goes beyond testing construct validity and showcases how the new scale(s) could be applied to investigate an unresolved issue in hypersexuality research: the relevance of orgasm frequency for PH ( Kafka, 1997; Reid & Grant, 2017; Štulhofer *et al*., 2016).

## Results

*Descriptive analyses.* A total of 1211 respondents from the general population completed the questionnaire and a total of 371 respondents seeking information on their level of hypersexuality completed the questionnaire via www.sekned.nl. Of the latter group of respondents, 187 (50.4%) indicated they had at least considered seeking help for problems experienced with "sex addiction", and these respondents were categorized as "PH"; the other part of this second sample, 184 respondents (49.6%), were categorized as "NH". In the GP sample, 30 participants (2.5%) indicated to have at least considered seeking help for sex addiction. Group averages for descriptive variables are reported in Table 1, together with the outcomes of t-tests for pairwise differences between the three subpopulations. It is to be noted that the NH group showed the highest sexual satisfaction and the PH group the lowest. There are no large differences in orgasm frequency between NH and PH; both groups report substantially higher orgasm frequencies than the GP sample. In the GP sample 1.2% of Pathos scores, 2.1% of HBI-19 scores and 0.4 % of CSBD-19 scores were above proposed cutoff values for these scales, indicating increased levels of sex addiction, hypersexual behavior or compulsive sexual behavior. In the NH sample 21.4 % of the Pathos scores, 34.8 % of the HBI-19 scores and 13.4 % of CSBD-19 scores were above cutoff. In the PH sample 49.5 % of the Pathos scores, 66.8 % of the HBI-19 scores and 28.8 % of CSBD-19 scores were above cutoff. We furthermore mention that for the SQ-48 Mood subscale, measuring depressive symptoms, 14.9 % of the GP scores, 29.4 % of NH scores and 54.3 % of PH scores were above the cutoff score. Significant differences in sexual orientation were found between the groups, though the effect sizes pertaining to these differences were moderate with the GP containing lower percentages of not as exclusively heterosexual identifying individuals (GP-NH: d = - 0.3; GP – NH: d = - 0.3; PH – NH: not significant).

**Table 1.** Average scores and effect sizes of significant differences on socio-demographic variables, sexual behavior and feelings and psychological symptoms for the GP, NH and PH subgroups.

	General Population (n=1211)	Non-problematic Hypersexuality (n=187)	Problematic Hypersexuality (n=184)	Effect size Cohen’s *d* of significant differences (GP-NH/ GP-PH/NH-PH)
Gender (w/m/o) *	592/618/1	77/109/1	39/144/1	X
Age	51.0 (17.3)	33.3(13.5)	36.1(12.8)	1.1/0.9/-0.2
Relationship not < 1 year > 1 year	23.9% 3.1% 73.0%	33.7% 16.8% 49.5%	24.1% 11.7% 64.2%	X
Agreement on sexual behavior between partners (1=Yes – 5=Not at all)	1.7(1.0)	2.3(1.4)	3.6(1.4)	-0.6/-1.8/-0.9
Kinseyscale (1=exclusively heterosexual – 7=exclusively homosexual).	1.5(1.4) Asexual: n=18	1.8(1.4) None asexual	1.9(1.4) Asexual: n=2	-0.3/-0.3/-
How often sex with partner **	2.9(1.5)	4.0(1.9)	3.3(1.6)	-0.7/-0.3/0.4
Number of different sexual partners in last six months	0.9(1.3)	3.2(4.6)	3.9(4.6)	-1.1/-1.4/-
Orgasm frequency ***	3.0(1.2)	4.7(1.2)	4.7(1.0)	-1.4/-1.5/-
Less/more orgasms than before (1=much less/3=equal/5=much more)	3.3(0.8)	3.4(1.0)	3.3(0.9)	-/-/-
Satisfied with sex life (1=Very – 5=Not at all)	3.3(1.2)	3.6(1.3)	2.9(1.2)	-0.3/0.4/0.6
Feel sexually addicted (1=Not at all – 5=Very)	1.3(0.6)	2.8(1.1)	3.3(0.9)	-2.1/-3.0/-0.6
Porn use (times per week) ****	2.1(1.5) No porn: n= 652 (53.8%)	4.6(2.1) No porn: n= 22 (12.0%)	5.1(1.8) No porn: n= 12 (6.4%)	-1.6/-2.1/-0.3
Currently help for PH	3	2	15	X
SQ-48 total	27.1(20.3)	39.0(27.4)	50.0(30.4)	-0.6/-1.1/-0.4
SQ-48 Mood	4.5(4.0)	6,3(5.7)	9.6(6.4)	-0.4/-1.2/-0.6
SES	12.3(4.1)	19.0(4.0)	19.3(3.7)	-1.6/-1.7/-
SIS1	8.5(3.1)	9.1(2.6)	10.0(3.0)	-0.2/-0.5/-0.3
SIS2	9.(3.6)	9.0(3.3)	9.5(2.9)	-/-/-
Pathos	8.9(2.9)	14.3(4.2)	17.4(3.8)	-1.8/-2.8/-0.8
HBI-19 total	26.0(9.1)	47.1(15.6)	59.4(15.3)	-2.1/-3.3/-0.8
CSBD-19 total	22.0(5.0)	35.8(12.2)	43.0(12.0)	-2.2/-3.3/-0.6

*In each of the three subsamples one person indicated to be of “other gender” (=”o”) than woman or man. **1=Not/2=two to five times/3=Once a month or a bit more/4=Once a week/5=A few times per week/6=Once a day/7=A few times per day (in past six months). ***1=No orgasm/2=Once a month or less/3=A few times per month/4=Once a week or more, but less than once a day/5=Once a day/6=Two to four times per day/7=More than four times per day (in past six months). ****1=No porn/2=Once a month or less/3=A few times per month/4=Once a week/5=Between 2 and 5 times per week/6=Once a day/7=Twice a day/8=More than twice a day.

*Item selection.* Of the 58 items addressing PH, 26 were selected to be included in the item bank(s). Of the 26 items, 8 (out of 14) came from the PHI, 2 (out of 6) from the Pathos, 10 (out of 19) from the HBI-19 and 6 (out of 19) from the CSBD-19 ( Table 2). Of note were the following results: The item intended to measure tolerance (PHI1: "I feel my longing for sex increases") showed a slightly higher average for NH than for PH indicating that both groups experienced a moderate increase in longing for sex; this item has not been included in the final list as it lacks power to differentiate between NH and PH. The item addressing withdrawal symptoms (PHI4: "When I stop with sex, I feel nervous and restless ") has not been selected as averages of NH and PH did not differ much (mean difference = 0.28, pooled SD = 1.32). Of the seven items pertaining to the Coping subscale of the HBI-19 only HBI18 ("I use sex as a way to try and help me deal with my problems") has been selected. On the other hand, six items addressing emotion dysregulation (3 of which address the use of sex as coping) from the newly developed items were selected. PHI14 ("I surrender to sex when I feel afraid") presented an edge case (mean difference = 0.58, pooled SD = 1.26), but based on its content it was selected. CSBD-19 items, in particular those of the Salience subscale (CSBD4, 5 and 6) and the Dissatisfaction subscale (CSBD10, 11 and 12), showed low differentiating power for NH and PH (mean difference between -.03 and 0.36, pooled SD between 1.02 and 1.08); none of these items were selected for the item banks. Of item HBI11 (discrimination parameter a = 3.7) and CSBD1 (a = 3.2) we chose to retain HBI11 as this item showed slightly better power to differentiate between PH and NH. Means and SD's for all selected items are presented in Table 2 for the GP, NH and PH sample.

**Table 2.** Item means and SDs for GP, NH and PH subgroups and IRT parameters per item (a: discrimination, b1–b4: thresholds, Hi: scalability and DIF: differential item functioning).

	GP M (SD)	NH M (SD)	PH M (SD)	a // b1/b2/b3/b4	H _i_	DIF
**Emotion Dysregulation-PH**						
Feeling stuck	1.6(0.9)	2.8(1.3)	3.7(1.1)	2.3 // 0.2/0.7/1.4/2.1	.60	
Sex when desperate	1.3(0.7)	2.2(1.4)	2.9(1.3)	3.3 // 0.6/1.0/1.6/2.1	.62	
Sex lowers self-esteem	1.3(0.7)	1.5(0.9)	2.6(1.3)	2.2 // 0.8/1.4/2.1/2.8	.55	Un ^1^
Think of sex when down	1.4(0.8)	2.7(1.3)	3.6(1.2)	2.9 // 0.2/0.7/1.4/2.1	.62	
After sex ashamed	1.3(0.7)	1.8(1.0)	2.8(1.2)	2.5 // 0.7/1.2/2.0/2.6	.57	NUn ^1^
Sex when frightened	1.2(0.5)	1.8(1.2)	2.4(1.3)	3.4 // 0.9/1.3/1.9/2.4	.61	
Depressed after sex	1.3(0.6)	1.6(1.0)	2.3(1.2)	2.5 // 0.8/1.5/2.3/2.8	.56	Un ^2^
Sex to handle problems	1.3(0.6)	2.2(1.0)	2.8(1.4)	2.9 // 0.7/1.2/1.7/2.3	.59	
Confused about sex	1.2(0.5)	1.9(1.0)	2.6(1.1)	2.2 // 0.9/1.6/2.3/-	.55	
**Negative Effects-PH**						
Ruin things with sex	1.3(0.6)	2.3(1.2)	3.2(1.2)	2.6 // 0.4/1.1/1.8/2.4	.65	
Empty after sex	1.5(0.8)	2.2(1.3)	3.0(1.2)	1.8 // 0.3/1.1/1.9/2.7	.55	
Controlled by sex	1.3(0.7)	2.8(1.3)	3.4(1.1)	3.4 // 0.3/0.9/1.5/2.0	.69	
Lost control over sex	1.4(0.8)	2.8(1.5)	3.8(1.1)	4.2 // 0.3/0.7/1.2/1.8	.72	
Continue sex while know one’ll regret it	1.2(0.6)	1.9(1.2)	3.2(1.2)	3.3 // 0.6/1.0/1.7/2.2	.66	
Discard important things in favor of sex	1.3(0.6)	2.1(1.2)	2.6(1.3)	2.3 // 0.7/1.3/2.1/2.6	.59	
Fail to change sex	1.4(0.8)	2.5(1.4)	3.7(1.1)	3.8 // 0.4/0.8/1.3/1.9	.70	
Had sex despite own values	1.3(0.6)	1.9(1.2)	2.8(1.4)	2.4 // 07/1.2/1.8/2.6	.59	
Not stopping sex even when risky	1.2(0.6)	2.5(1.5)	3.2(1.4)	3.6 // 0.6/1.0/1.4/2.0	.68	
Sex leads one in wrong direction	1.2(0.6)	2.0(1.2)	3.2(1.2)	4.0 // 0.7/1.1/1.6/2.1	.70	
Sex controls one’s life	1.2(0.5)	2.4(1.3)	3.2(1.2)	3.9 // 0.6/1.1/1.6/2.1	.70	
Sex stronger than self-discipline	1.4(0.8)	3.1(1.4)	3.8(1.1)	4.2 // 0.2/0.7/1.2/1.7	.73	
Did not control sex long	1.1(0.4)	2.1(1.1)	2.6(1.0)	3.8 // 0.8/1.4/1.9/-	.69	
Failed to limit sex	1.1(0.4)	2.0(1.1)	2.5(1.0)	3.2 // 0.9/1.4/2.0/-	.65	
Negative changes by sex	1.1(0.4)	1.6(0.9)	2.3(1.0)	3.7 // 1.1/1.6/2.1/-	.68	
Negative impact of sex on relations	1.1(0.4)	1.7(1.0)	2.4(1.1)	2.9 // 1.0/1.6/2.1/-	.63	
No healthy sex anymore	1.1(0.4)	1.5(0.9)	2.2(1.0)	2.8 // 1.1/1.7/2.3/-	.61	

Un ^1^: Uniform DIF for the NH – PH contrast (for Emotion Dysregulation-PH) NUn ^1^: Non-Uniform DIF for the NH – PH contrast (for Emotion Dysregulation-PH) Un ^2^: Uniform DIF for the GP – (NH+PH) contrast (for Emotion Dysregulation-PH)

**Figure 1.**
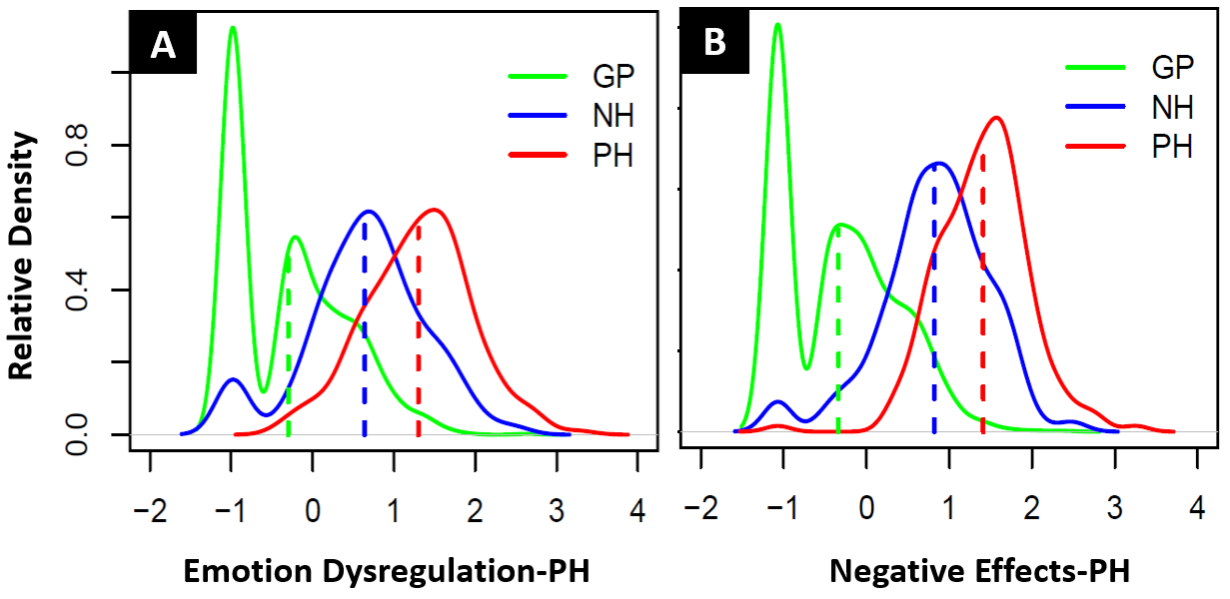
( **A**) Relative density distribution of GP, NH and PH latent trait scores for the Emotion Dysregulation-PH and ( **B**) Relative density distribution of GP, NH and PH latent trait scores for the Negative Effects-PH scale.

*Structural validity.* The 26 items that were selected for the initial item bank could – following the factor structure in Van Tuijl *et al*., 2020 – be divided into two factors: 1) “Emotion Dysregulation-PH”, consisting of 9 items representing the distressing emotional patterns co-occuring with hypersexual preoccupation; and 2) “Negative Effects-PH”, consisting of 17 items representing the negative consequences of patterns of hypersexual thoughts and behavior. Items that might have pertained to other factors, for instance concerning tolerance or preoccupation (the “Sexual Desire” factor in Van Tuijl *et al*., 2020) or items concerning social aspects (e.g. “I would rather not tell those close to me how I behave sexually“) were not selected from the original list of 58 items as these items did not pass initial item selection. A two-factor model fitted the data well (CFI: 0.96; TLI: 0.96; RMSEA: 0.07; SRMR: 0.05). Internal consistency for both scales was sufficient. Emotion Dysregulation-PH showed an ECV of .73, a hierarchical omega of .80 and an Eigenvalue ratio of 9:1. Negative Effects-PH showed an ECV of .89, a hierarchical omega of .92 and an Eigenvalue ratio of 30:1. Both factors met the assumption of unidimensionality with scaled fit measures for Emotion Dysregulation-PH (CFI: 0.96; TLI: 0.96; RMSEA: 0.13; SRMR: 0.07) and for Negative Effects-PH (CFI: 0.99; TLI: 0.99; RMSEA: 0.07; SRMR: 0.03) showing acceptable to good fit. Only the RMSEA-scaled fit for Emotion Dysregulation-PH showed slightly higher values than .10, but this is not unusual in PROMIS or similar validation studies ( Cook *et al*., 2009). The assumption of local independence was met for both scales as no item pairs showed residual correlations > .2. The assumption of monotonicity (H _i_ > .3, H > .5) was met as both scales had no single items with H _i_ < .54 and overall monotonicity of the scales was .59 for Emotion Dysregulation-PH and .66 for Negative Effects-PH. Item monotonicity values are presented in Table 2. A graded response model was fitted to each of the scales separately. Item discrimination and threshold parameters are presented in Table 2. Discrimination parameters ranged from 2.24 to 3.38 for Emotion Dysregulation-PH and from 1.81 to 4.24 for Negative Effects-PH. Item discrimination and threshold values are presented in Table 2. The distribution of the latent trait scores of the GP, NH and PH groups for both scales are displayed in relative densities plots in Figure 1. Item characteristic curves have been visually inspected for the combination of the NH-PH subgroup, and for 24 out of 26 items the second response category was completely overlapped by adjacent response categories; there was almost no overlap of the second response category in the GP sample (see supplemental material). No items on the two scales displayed item misfit (S-X ^2^ < .001).

**Figure 2.**
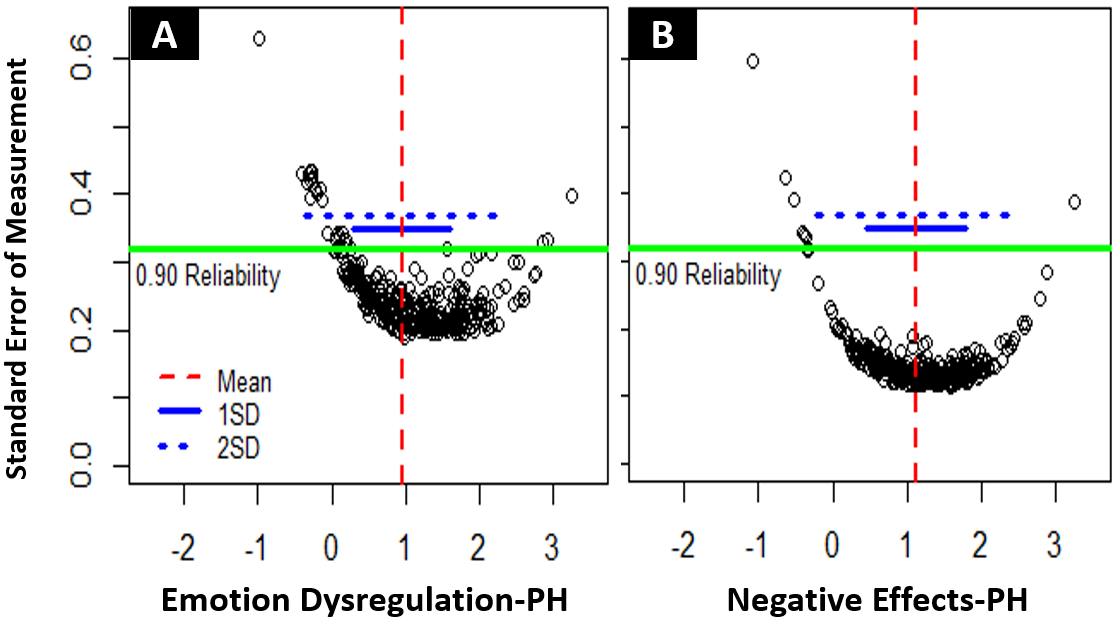
( **A**) Reliability of measurements for Emotion Dysregulation-PH. ( **B**) Reliability of measurements for Negative Effects-PH. Each dot represents an individual measurement on the latent trait scale – below the green line scores are measured with .9 (SEM = .316) or higher reliability.

*Latent trait reliability.* We used standard error of measurement (SEM) – with SEM < .316 representing reliability of measurements of .9 or higher – to assess reliability over the range of the latent traits of the two scales. With Emotion Dysregulation-PH, 83.8 % of NH-PH participants was reliably measured, with a latent trait mean of 0.95 (SD = 0.80) and an average SEM of 0.27. With Emotion Dysregulation-PH, only 31.4 % of the GP participants was reliably measured (M _GP_ = -.29, SD _GP_ = 0.63, average SEM _GP_ = 0.44). Only considering the NH-PH sample, latent trait levels were reliably measured approximately from 1.5 SD below the mean to 2.5 SD above the mean of 0.95 (see Figure 2a). With Negative Effects-PH, 95.7 % of NH-PH participants was reliably measured, with a latent trait mean of 1.12 (SD = 0.68) and an average SEM of 0.16. With Negative Effects-PH, only 48.6 % of the GP participants was reliably measured (M _GP_ = -.34, SD _GP_ = 0.69, average SEM _GP_ = 0.37). Only considering the NH-PH sample, latent trait levels were reliably measured approximately from 2 SD below the mean to 3 SD above the mean of 1.12 (see Figure 2b). Extensive floor effects were observed for both scales in the GP sample, with 42.4 % of Emotion Dysregulation-PH scores and 37.7 % of Negative Effects-PH scores representing the absolute minimum.

*Differential Item Functioning (DIF).* We performed DIF-tests on four different contrasts for the Emotion Dysregulation-PH and Negative Effects-PH scale. No DIF was detected for gender (women compared to men) and sexual orientation (exclusively heterosexual compared to not exclusively heterosexual) for Emotion Dysregulation-PH. For this scale, we detected uniform DIF for the NH-PH contrast for the item “Sex gives me a bad feeling about myself“ with the PH group showing increased probability to score higher given the same underlying latent trait score. Non-uniform DIF for the NH-PH contrast was detected for the item “After sex I feel ashamed“. Furthermore, we detected uniform DIF for Emotion Dysregulation-PH for the GP-(NH+PH) contrast for the item “When you have sex, do you feel depressed afterwards?“ with the NH+PH group showing increased probability to score higher given the same underlying latent trait score. For Negative Effects-PH no DIF was detected for any of the four contrasts.

*Interpretability.* Average sum scores and SD’s for GP, NH and PH are given for women and men in Table 3 for the Emotion Dysregulation-PH and Negative Effects-PH scale. The correlation between latent trait scores and sum scores was .93 (95% CI: .93 - .94) for Emotion Dysregulation-PH and .91 (95% CI: .90 - .92) for Negative Effects-PH, showing that sum scores represent an adequate, though not perfect, measure of the underlying latent trait ( McNeish & Wolf, 2020). As sum scores are currently the sole option for applied clinical practice to work with, from hereon we present analyses using sum scores. From Table 3 it can be gathered that women scored lower than men on both scales in the GP sample while they scored higher than men on both scales in the NH and PH samples – though sample size for women was rather low in the PH sample. As DIF for gender was absent, this indicates that the women who participated in the web-based survey experienced higher levels of emotion dysregulation and negative effects due to PH than men. The AUC regarding correct classification of NH and PH in the NH + PH subsample was .75 (95% CI: .70 - .80) for Emotion Dysregulation-PH and .76 (95% CI: .71 - .81) for Negative Effects-PH, showing acceptable power to differentiate between NH and PH for both scales. To check differences in scale scores for different sexual orientations (Kinsey scale, 1 = exclusively heterosexual – 7 = exclusively homosexual, 8 = asexual (n=19)), first we performed separate regression analyses for each group and each scale. The six analyses all showed that an increase in Kinsey scale scores – moving from hetero- to homosexual – was associated with an increase in Emotion Dysregulation-PH or Negative Effects-PH. However, sexual orientation only explained 0.3 tot 3.9 % of the variance of the outcome variable. For the GP, PH and NH group the effects were similar for both scales. In the supplemental material we report in more detail on sexual orientation and present interaction plots to illustrate that effects of Kinsey scale scores on the two scales are similar for the three groups. 

**Table 3.** Overall and gender means + SD’s per scale for GP, NH and PH subsamples.

	General Population -overall M (SD) - (women n=592/men n=618, n=1 other)	Non-problematic Hypersexuality - overall M (SD) - (women n=77/men n=109, n=1 other)	Problematic Hypersexuality - overall M (SD) - (women n=39/men n=143, n=1 other)
Emotion Dysregulation-PH	M=11.7 (SD=4.0) w-11.0 (3.0) / m-12.4 (4.7)	M=18.4 (SD=6.9) w-21.2 (7.7) / m-16.5 (5.7)	M=25.4 (SD=7.7) w-29.3 (7.6) / m-24.3 (7.5)
Negative Effects-PH	M=21.2 (SD=6.6) w-19.4 (4.1) / m-23.0 (8.4)	M=37.5 (SD=13.3) w-41.0 (14.4)/ m-34.8 (11.8)	M=53.1 (SD=13.8) w-53.5 (14.1) / m-50.1 (12.9)

**Figure 3.**
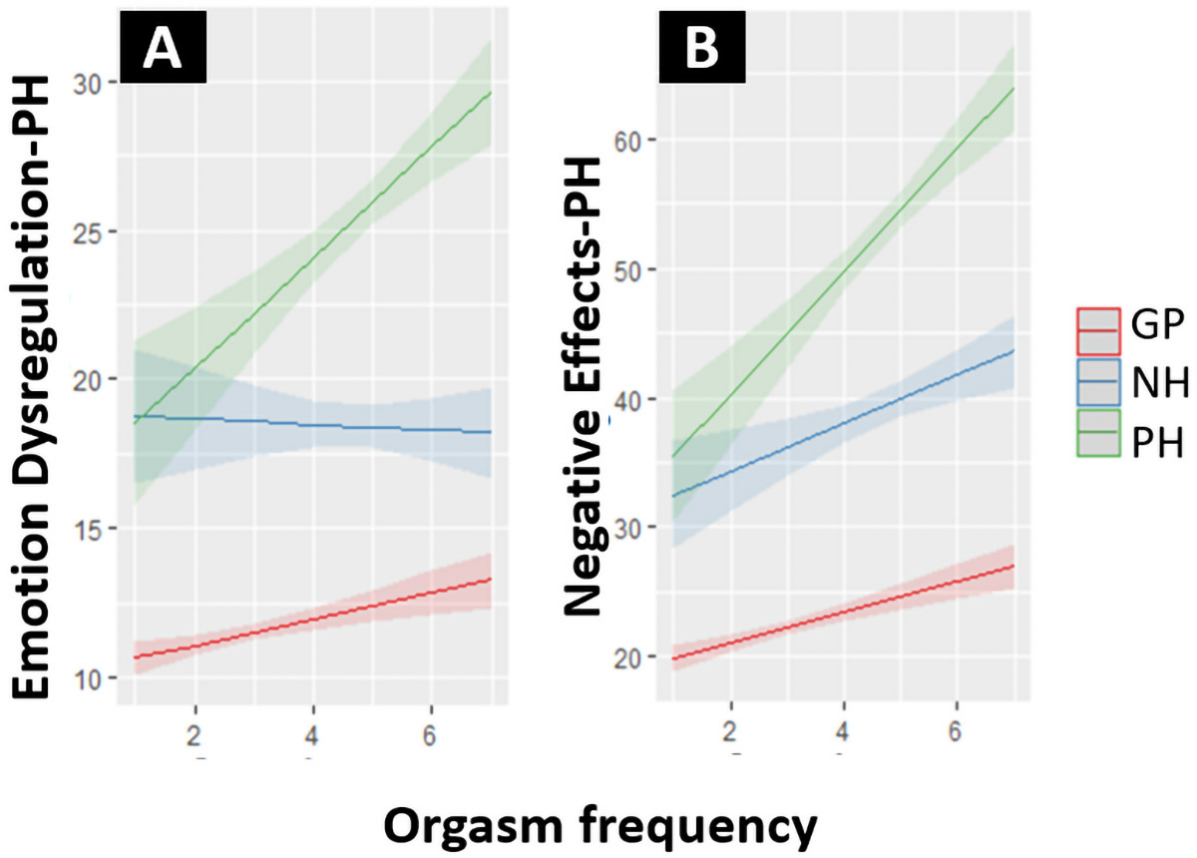
( **A**) Effect of orgasm frequency on Emotion Dysregulation-PH for the GP, NH and PH subpopulation. ( **B**) Effect of orgasm frequency on Negative Effects-PH for the GP, NH and PH subpopulation. Note that the orgasm frequency scale (1 – 7) represents “In the past six months. No orgasm (1)/Once a month or less (2)/A few times per month (3)/Once a week or more, but less than once a day (4)/Once a day (5)/Two to four times per day (6)/More than four times per day (7)”.

*Construct validity.* In the GP sample the PH group (n=30; M _ED_ = 20.0; SD _ED_ = 7.6; M _NE_ = 37.3; SD _NE_ = 15.0) scored significantly higher on Emotion Dysregulation-PH and Negative Effects-PH than the rest of the sample (n=1181; M _ED_ = 11.5; SD _ED_ = 3.6; *t*(29.3) = 6.06; *p* < .001; *d* = 2.3; M _NE_ = 20.8; SD _NE_ = 5.7; *t(*29.2) = 6.02; *p* < .001; *d* = 2.7). In the GP sample the PH group (for M and SD see previous sentence) scored significantly higher on both scales than participants who reported to have had at least one orgasm per day or more in the past six months (n=65; M _ED_ = 12.9; SD _ED_ = 4,7; *t*_ED_(39.3) = 4.67; *p*_ED_ < .001; *d*_ED_ = 1.2; M _NE_ = 23.0; SD _NE_ = 7.4; *t*_NE_(35.8) = 4.94; *p*_NE_ < .001; *d*_NE_ = 1.4). For the GP sample the correlation between the Mood subscale of the SQ-48 and Emotion Dysregulation-PH was weak: .33 (95% CI: .28 - .38) and the correlation between Mood and Negative Effects-PH was very weak: .19 (95% CI: .13 - .24). For the NH-PH sample the correlation between Mood and Emotion Dysregulation-PH was strong: .67 (95% CI: .62 - .73) and between Mood and Negative Effects-PH it was moderate: .49 (95% CI: .41 - .56). The effect of orgasm frequency on Emotion Dysregulation-PH and on Negative Effects-PH significantly differed between groups, with the strongest positive effects visible for the PH group, as shown in Figure 3. For the NH group there was no effect of orgasm frequency on Emotion Dysregulation-PH. Effects for the GP sample were small, with increases in orgasm frequency only leading to slight increases in the outcomes on both scales. All hypotheses specified to test construct validity were supported.

## Discussion

This is the first study that investigated a combination of indicators stemming from three diverging diagnostic perspectives on problematic hypersexuality ( Montgomery-Graham, 2017), and the first study that used IRT to develop item banks for PH. Our use of extensive samples of NH and PH participants further allowed us to pursue our twofold research goals: 1) psychometric development and validation of item banks for PH; and 2) update the preliminary definition of PH.

### Development and validation of item banks for PH

An item bank has been developed for an Emotion Dysregulation-PH scale, measuring the distressing emotional patterns co-occurring with hypersexual preoccupation. An item bank has also been developed for a Negative Effects-PH scale, measuring the negative consequences of patterns of hypersexual thoughts and behavior. The item banks for these scales displayed sufficient structural validity, reliability, interpretability and construct validity in the target population that consists of NH and PH individuals. For the Emotion Dysregulation-PH scale DIF was detected for a total of three items, for different contrasts. DIF was small in these cases and did not gravely impact the total scale score. For the Negative Effects-PH scale no DIF was detected. No DIF was detected for women and men for both scales. As women scored higher on the scales, this implies that women participating in the NH and PH group experienced more emotion dysregulation and negative effects due to hypersexuality. No DIF was detected for sexual orientation. For future development of the item banks, items first need to be homogenized (e.g. all questions turned into statements) and the number of response categories needs to be brought back to four. This study showed that the response category “Rarely” was relatively seldomly chosen in the NH + PH sample. We note that the newly developed scales are less valid for measurements of PH in the general population as large floor effects occurred in the GP sample. As the new scales are partly based on previously developed measurement instruments, it is likely that these previously developed instruments are less valid in the GP as well, at least for the lower ranges of PH in the general population. In order to make the scales more reliable in the GP, items could be added that can differentiate between levels in the lower ranges of the latent traits (low to absent hypersexuality). It is questionable if this is useful, however, as the scales will be specifically used in situations where it is important to differentiate PH from other conditions that closely resemble it but are nonetheless different from it (e.g. NH). Given the low base rate of PH, we advise caution when using the new scales for diagnostic or screening purposes in the general population. In such research, the scales should only be used to assess prevalence of PH for general overview purposes and not for the identification of PH in individual participants. Further development of the item banks is planned, for instance by validating short forms. Before such research is undertaken, we need to update our preliminary definition of PH and bring it in line with the results of the current study.

### Updating the definition of Problematic Hypersexuality

This study also aimed to investigate the preliminary, testable definition of PH and determine if the stable characteristics mentioned in the definition show sufficient cue-validity – sufficient uniqueness as indicator of PH. Results in this regard are mixed. Three remarks can be made. Firstly, we remark that the characteristic “preoccupation with sex” was not cue-valid for PH. Similar levels of preoccupation with sex are visible in NH, shown by the similar scores of the NH and PH group on the Salience scale from the CSBD-19 ( Böthe *et al*., 2020) and by a lack of difference in orgasm frequency between NH and PH. Note that this result does not contradict that preoccupation with sex is a necessary condition and driving force for PH but only stresses that preoccupation with sex is not unique to PH. Secondly, we remark that most items from the HBI-19 Coping scale ( Reid *et al*., 2011) lacked cue-validity in the NH-PH target population. We had expected this, based on previous research showing that sex is used to cope with negative feelings by a significant part of the general population as well, which defies the uniqueness of this characteristic to PH ( van Tuijl *et al*., 2022b). Therefore, we added more extreme items expressing the use of sex as coping (e.g. “I have sex to escape feelings of despair”). We also added items that expressed the vicious circle of emotion dysregulation (e.g. “I feel stuck in my sexual behavior”); these items also relate to coping mechanisms in PH ( Adams & Robinson, 2001; Rahm-Knigge *et al*., 2023; van Zessen, 2013). Previously we defined emotion dysregulation as a driving force of PH but had not conceptualized it as a measurable stable trait characteristic. The current study shows that emotion dysregulation can also be measured as a cue-valid stable trait of PH. Thirdly, we remark that the PH characteristics of tolerance (escalating desire for sex) and withdrawal symptoms (feel restless when stopping sex) did not show sufficient cue-validity. However, previous research showed that in PH a gradual escalation of sexual frequency was experienced ( Reid *et al*., 2012) and that tolerance and withdrawal were associated with CSBD ( Lewczuk *et al*., 2022). However, the operationalization of withdrawal in the latter study (e.g. “frequent sexual thoughts that were difficult to stop”) might rather express sexual preoccupation, and in both studies cue-validity of characteristics was not tested as no relevant subpopulations for comparisons were included. In future studies we aim to test tolerance and withdrawal with different items. For the time being, we retain tolerance and withdrawal as possible cue-valid indicators for PH, in need of further investigation, following Carnes *et al*. (2014) who found high levels of both in PH-samples. In conclusion we remark that the preliminary definition of PH we proposed needed to be updated in order to align with the results of the current study. We have done so; the updated definition can be found at https://psycore.one/ProblematicHypersexuality_7qfdty7g

### Towards a diagnosis for Problematic Hypersexuality?

Our results showed that there are many differences between the NH and PH subpopulations. Nonetheless, we still observed a large overlap suggesting that currently even the most cue-valid set of PH indicators – the set we found – runs the risk of yielding large percentages of false positives ( Wakefield, 2012). The NH sample, furthermore, showed higher levels than the GP sample for overall psychological distress symptoms and for depressive symptoms, though NH scores were well below PH levels for these measures. Apparently, also in NH increased levels of distress are experienced. All these results illustrate that questionnaire-based failsafe categorization of hypersexual individuals as problematic might not be justifiable at the moment. This is in particular important given there are numerous people who “diagnose” themselves as sex or pornography addicted and might experience negative consequences from this ( Prause & Binnie, 2023). Therefore, the item banks we developed and validated should not be used for diagnosis, but should only be used as an instrument to preliminarily gauge the nature of someone’s hypersexual preoccupation. If used to self-assess, a doctor’s or sexologist’s can be needed to complement the self-assessment. Despite these caveats, our study showed that a differentiation between NH and PH is viable and useful to determine the indicators that are most unique to PH. In line with our results, previous research also illustrated a differentiation between NH and PH. For instance, Van Zessen (1995) showed in a large qualitative study in hypersexual participants, that those focusing on positive incentives to engage in sexual activity (contact seeking) experienced less negative effects than other hypersexual participants for whom sex had become a way to divert feelings of emptiness and low self-esteem ( Van Zessen, 2013). Furthermore, in line with our results, Štulhofer *et al*. (2016) revisited the claim that hypersexuality is similar to high sexual desire and found significant differences between a (problematic) hypersexual and a high sexual desire sample, especially with regard to characteristics expressing emotional turmoil and negative consequences. In hypersexuality – we suggest – differentiation between PH and NH is possible, but categorization remains problematic. Kraus *et al*. (2018) introduced the CSBD diagnosis – an improvement over the ICD-10 diagnosis of Excessive sexual drive ( World Health Organization, 2016, code F52.7) – and stated in their guidelines that guilt and shame in themselves are not sufficient to diagnose the condition. However, it might be exactly such indicators of emotion dysregulation that can provide important information on the amount and characteristics of problems experienced due to hypersexuality ( Grubbs *et al*., 2019). We agree that guilt and shame are problematic as diagnostic criteria, but our study showed that they can nonetheless be used to differentiate between levels of problems experienced due to hypersexuality. Differentiation, however, will not be perfect as there is also extensive overlap between NH and PH in emotion dysregulation. Therefore, and for the other reasons mentioned above, we propose PH not as a diagnosis but as a comprehensive construct that that can offer a detailed description – but not classifications – to all who experience – to a greater or lesser extent – distress related to hypersexuality.

### Limitations

A number of limitations of this study need to be mentioned. One limitation concerns the gold standard that was used to discern NH from PH. The NH sample might partly consist of participants who do need help but are not ready to admit this yet. On the other hand, the PH sample might partly consist of people who are just in moral quandaries about their sexual feelings and behavior ( Cantor *et al*., 2013). A more optimal gold standard could be established by using clinical interviews (e.g. Reid *et al*., 2012) of NH and PH individuals to determine if clinical help is recommended. Another limitation of this study concerns the absence of repeated measurements in the NH and PH subpopulations. This would have provided a measure of responsiveness, the extent in which the instrument is capable of measuring minimal important change over time ( Terwee *et al*., 2007). Responsiveness is important in determining the evaluative value of an instrument, and can also provide an indication of how the instrument can be used to measure the severity of PH ( Reid, 2015). Therefore, future research on the item banks should also include repeated measurements in NH and PH samples. A further limitation of this study is that due to its psychometric objective and lack of space, only a limited investigation of similarities and differences between women and men have been undertaken– and also that in the PH sample much less women participated than men. Nonetheless, preliminary results suggest that participating women in the NH and PH group experienced more distress due to hypersexuality and this calls for further analyses in future studies investigating this sample. Regarding sexual orientation, we like to mention that the Kinsey scale might not be the most optimal instrument to gather information with as it sees bisexuality not as a separate identity but as the middle ground between hetero- and homosexuality. Furthermore, our follow-up analyses showed that in all three groups – general population, PH and NH – moving from hetero- to homosexuality was associated with increased scores of Emotion Dysregulation-PH and Negative Effects-PH. Though effects were small, this can implicate bias in the questionnaire signaling out more sexually open lifestyles. DIF, however, was absent for sexual orientation. A final limitation we need to mention concerns content and cue-validity. As our results showed, there is a large overlap between NH and PH subpopulations with regard to the newly developed scales. More content and cue-valid items need to be developed to improve differentiation between both groups. In this study we used quantitative analyses to test new items we developed ourselves. In order to find more cue-valid items for PH, we suggest using cognitive interviews - together with other qualitative methods - in relevant subpopulations. Qualitative methods can secure valid item content and, in combination with quantitative methods, can help establish a more extensive cue-valid item bank for PH.

## Conclusion

We demonstrated many advantages of IRT analyses for the development and validation of item banks to measure PH. Item information and the use of an NH – PH subsample made a more nuanced assessment possible of the content and cue-validity of items than with previous validation methods. Two item banks were developed and validated – Emotion Dysregulation-PH and Negative Effects-PH, both showing good psychometric features. The present study was used to update our preliminary definition of PH. Furthermore, this study illustrated that a categorical distinction between NH and PH is still hard to make – based on a questionnaire – and we therefore abstain from suggesting using the developed item banks to decide on if someone “has or doesn’t have” PH. Notwithstanding, the item banks we developed can well supplement the assessment of level of PH in clients seeking treatment for distress related to hypersexuality.

## Data Availability

Open Science Framework: IRT validation of item banks to measure problematic hypersexuality https://doi.org/10.17605/OSF.IO/8X7VZ ( van Tuijl *et al*., 2023b) This project contains the following underlying data and analysis script (R code) and Supplemental Material: DataPHDS_1a.sav (The data of 1579 participants/rows and 146 variables/columns – these are the data that are used in the analyses described. Note that the variable “Age” and three participants who had indicated to be of “other” gender have been removed from the data in order to avoid recognition of these participants.) PHDS total.html (Description of the methods and results as presented in the article; also, the R-code and output of all reported analyses are included in this file.) Supplemental material to Initial development and validation of item banks to measure problematic hypersexuality.doc (Supplemental Material to version 2 of the manuscript). Data are available under the terms of the Creative Commons Zero "No rights reserved" data waiver (CC0 1.0 Public domain dedication).
