# Supplementary material for: Initial development and validation of item banks to measure problematic hypersexuality
Source: Open Res Eur. 2024 Nov 22;3:129. Originally published 2023 Aug 24. [Version 3] doi: 10.12688/openreseurope.16131.3 (PMC11306953; doi:10.12688/openreseurope.16131.3)
Supplement: Peer review history

# Reviewer report: Reviewer response for version 3

**DOI:** 10.21956/openreseurope.20268.r47793

**Published:** 2025-01-06

**Authors:** Philipp E Sischka

**Affiliations:** 1 University of Luxembourg, Esch-sur-Alzette, Luxembourg

Thank you for the opportunity to review this manuscript, which aims to develop and validate item banks to measure problematic hypersexuality. Since my primary area of expertise is not sexuality, my review will focus on the methodological aspects of the manuscript. To begin with, I would like to say that I found the manuscript to be of a high standard and well done. My comments mainly address areas of ambiguity. 

 Introduction

 The advantages of IRT over factor analysis reads a bit abstract for researchers not familiar with IRT. You could also add the following advantages: First, IRT models typically exploit all available data, whereas CFA only uses summary statistics (e.g., Wirth & Edwards, 2007). Because of that, (multidimensional) IRT analysis is also called full-information item factor analysis (e.g., Cai et al., 2011). Second, IRT models take the ordinal nature of the items into account (although in CFA this can be done for instance via WLSMV estimator) and have a stronger focus on understanding the performance of each individual items (Houts et al., 2022). Third, reliability coefficients based on CFA/classical test theory (e.g., Cronbach’s α) implicitly assume that the standard error of measurement is uniform across the latent variable continuum. In contrast, by incorporating item characteristics IRT models acknowledge that reliability of person scores across different levels of the latent variable may differ as well and are able to derive a conditional reliability analogue (O’Connor, 2018a/b; Markon, 2018), i.e., the reliability across the latent continuum (Houts et al., 2022).

 Statistical analysis

 Please provide an effect size measure for the associations between the categorical variables (e.g., gender and the subgroups), such as Cramer’s V.

 Results

 Please provide the item selection statistics (i.e., mean and pooled SD) for all 58 items (not just the 26 selected) to clarify the decision-making process behind item selection. This information does not need to be included in the manuscript but could be provided in the supplemental material.

 I found it challenging to understand which factor models were actually tested. You wrote that “[a] two-factor model fitted the data well.” Did you test a correlated two-factor model, a bifactor model with a general factor and two specific factors (i.e., emotion dysregulation and negative effects PH factors), or two separate bifactor models (i.e., one with the items for emotion dysregulation PH and one with the items for negative effects PH)? If so, which specific factors were included in the calculation of the ECV? Including a graphical representation would greatly enhance the clarity and detail of the tested factor models.

 You wrote: “A graded response model was fitted to each of the scales separately.” If this was a unidimensional model (as mirt is also capable of calculating a bifactor GRM), please specify this.

 You wrote: “No items on the two scales displayed item misfit (S-X² < .001).” Shouldn’t the 'greater than sign' be the other way around?

 Figure 1: Please include a note explaining what the dashed lines represent (I assume they indicate the mean within these subpopulations, but this should be explicitly stated).

 Would it be possible to include an effect size measure for differential item/test functioning (see, e.g., Chalmers, 2018)? Some of these measures are also available in the mirt package (e.g., the function mirt::DRF).

 You wrote: “As DIF for gender was absent, this indicates...” Please keep in mind that a non-significant DIF effect does not necessarily indicate the absence of a DIF effect in the population (e.g., Wasserstein et al., 2019).

 Minor points

- The information that the SQ-48 consists of nine subscales is directly repeated: “The SQ-48 is a nine-scale, 48-item instrument measuring psychological symptoms, distress and vitality in the past week. It consists of nine subscales.”
- Please specify the used R version (just type R.Version() in the console) for the sake of reproducibility.
- Remove the second “Secondly” in the following sentence: “Secondly, Secondly, we checked DIF for sexual orientation”.
- Please provide sample items for the Sexual Inhibition/Sexual Excitation Scales.

References

 [Ref 1], [Ref 2], [Ref 3], [Ref 4], [Ref 5], [Ref 6], [Ref 7] and [Ref 8].

 All other mentioned references here are listed in the manuscript.

If applicable, is the statistical analysis and its interpretation appropriate?

Yes

Is the study design appropriate and is the work technically sound?

Yes

Is the work clearly and accurately presented and does it engage with the current literature?

Yes

Are the conclusions drawn adequately supported by the results?

Yes

Are sufficient details of methods and analysis provided to allow replication by others?

Partly

Are all the source data and materials underlying the results available?

Partly

Reviewer Expertise:

Item response theory, latent variable modeling, questionnaire development, scale validation

I confirm that I have read this submission and believe that I have an appropriate level of expertise to confirm that it is of an acceptable scientific standard, however I have significant reservations, as outlined above.

## References

[1] : Generalized full-information item bifactor analysis. *Psychol Methods* .2011;16(3) : 10.1037/a0023350 221-48 10.1037/a0023350 21534682 PMC3150629

[2] : Model-Based Measures for Detecting and Quantifying Response Bias. *Psychometrika* .2018;83(3) : 10.1007/s11336-018-9626-9 696-732 10.1007/s11336-018-9626-9 29907891

[3] : Overview of Modern Measurement Theory and Examples of Its Use to Measure Execution Function in Children. *Journal of Pediatric Neuropsychology* .2022;8(1) : 10.1007/s40817-021-00117-7 1-14 10.1007/s40817-021-00117-7

[4] : Reconciling information and reliability in scaling local measurement precision: A comment on O'Connor (2017). *Psychol Assess* .2018;30(8) : 10.1037/pas0000548 1004-1009 10.1037/pas0000548 30070573

[5] : An illustration of the effects of fluctuations in test information on measurement error, the attenuation of effect sizes, and diagnostic reliability. *Psychol Assess* .2018;30(8) : 10.1037/pas0000471 991-1003 10.1037/pas0000471 28301193

[6] : Clarifications regarding test information and reliability, and new methods for estimating attenuation due to measurement error: Reply to Markon (2018). *Psychol Assess* .2018;30(8) : 10.1037/pas0000632 1010-1012 10.1037/pas0000632 30070574

[7] : Moving to a World Beyond “p < 0.05”. *The American Statistician* .2019;73(sup1) : 10.1080/00031305.2019.1583913 1-19 10.1080/00031305.2019.1583913

[8] : Item factor analysis: current approaches and future directions. *Psychol Methods* .2007;12(1) : 10.1037/1082-989X.12.1.58 58-79 10.1037/1082-989X.12.1.58 17402812 PMC3162326

# Reviewer report: Reviewer response for version 3

**DOI:** 10.21956/openreseurope.20268.r47798

**Published:** 2024-12-13

**Authors:** Wei Wang

**Affiliations:** 1 Norwegian University of Science and Technology, Trondheim, Norway

This is a nonclinical study conducted in 371 participants, but the term "problematic hypersexuality" is not well defined, through the participant selection and through the results presentation.

 1) There are some structural-validated hypersexuality scales available in the literature but not mentioned in the manuscript, for instance, the one in Chinese culture (BMC Psychiatry 2021). Therefore, the current study is not the first study relating to hypersexuality, whether regarding the measure or the diagnosis.

 2) The tested items measuring hypersexuality are not exhaustive, especially those relating to clinical aspect. For instance, the sexual impulse control and the sexual obsessive-compulsive aspects.

 3) Compared to those published questionnaires measuring hypersexuality, the current study only identified emotional dysregulation and negative effects. Actually, the negative Impact and emotional coping has been covered by previous publication, the impulsive behavior and OCD trait were less mentioned in the manuscript. Meanwhile the afterwards regret was not identified either. Even the current study's negative effects included the thoughts and behavior, much information has been lost in the current design.

 4) IRT is a very nice tool to discover the related information of a measure, but the current study missed much information instead. The authors might first retrieve the latent factors inside the questionnaire answer-using the exploratory factor analysis, that will make the latent factor coming out.

 5) When selecting participants of the problematic hypersexuality, the authors might apply some criteria to participants (clinical-diagnostic, for emotional, behavioral or interpersonal). The "at least 0,5 x SD" is not enough.

 6) The might also be sex (gender) differences of the hypersexuality measure, where the current study can address.

 7) Please always provide the raw data (in groups, the mean and SD for each scale) when presenting the related Results.

 8) Are there any ongoing emotional problems in participants with problematic hypersexuality? If yes, how they are related to the measure?

 9) In the Discussion section, some paragraphs were written but without proper references.

If applicable, is the statistical analysis and its interpretation appropriate?

Partly

Is the study design appropriate and is the work technically sound?

Partly

Is the work clearly and accurately presented and does it engage with the current literature?

Partly

Are the conclusions drawn adequately supported by the results?

Partly

Are sufficient details of methods and analysis provided to allow replication by others?

Partly

Are all the source data and materials underlying the results available?

Partly

Reviewer Expertise:

Personality trait, personality disorder, bipolar disorder, neurophysiology, cerebral imaging, cultural issues, clinical psychiatry, clinical psychology

I confirm that I have read this submission and believe that I have an appropriate level of expertise to confirm that it is of an acceptable scientific standard, however I have significant reservations, as outlined above.

# Reviewer report: Reviewer response for version 2

**DOI:** 10.21956/openreseurope.19559.r42002

**Published:** 2024-08-07

**Authors:** Kai Jonas

**Affiliations:** 1 Department of Work and Social Psychology, Maastricht University, Maastricht, Limburg, The Netherlands

I would like to thank the authors for their revisions. I believe that the manuscript has gained much in clarity and the research aim is clearer now. If I may suggest one minor addition: under Limitations and future research, the authors point to a gender difference in perceived burden and suggest future research - which I fully agree with - but I would add that the gender distribution, especially in the PH group, is very uneven and with n=39 for women rather small. Thus there may also be statistical limitations at play.

If applicable, is the statistical analysis and its interpretation appropriate?

Partly

Is the study design appropriate and is the work technically sound?

Partly

Is the work clearly and accurately presented and does it engage with the current literature?

Partly

Are the conclusions drawn adequately supported by the results?

Yes

Are sufficient details of methods and analysis provided to allow replication by others?

Yes

Are all the source data and materials underlying the results available?

Partly

Reviewer Expertise:

sexual wellbeing, sexual health, self-regulation, substance use, sexual identities, discrimination

I confirm that I have read this submission and believe that I have an appropriate level of expertise to confirm that it is of an acceptable scientific standard.

# Response

**Published:** 2024-10-09

**Authors:** Piet van Tuijl

**Affiliations:** Psychology, Open University, the Netherlands, Heerlen, Limburg, The Netherlands

Dear Professor Kai Jonas, We added two sentences to the article, expressing only a small sample of women with PH participated. Thank you for your review and commentary, Kind regards, Piet van Tuijl

# Reviewer report: Reviewer response for version 1

**DOI:** 10.21956/openreseurope.17414.r38460

**Published:** 2024-04-19

**Authors:** Kai Jonas

**Affiliations:** 1 Department of Work and Social Psychology, Maastricht University, Maastricht, Limburg, The Netherlands

The authors are presenting a MS proposing an improved way to measure problematic hypersexuality, using an item response theory approach. I would like the share the following comments with the authors:

 Abstract: I would remove the information about the percentage of those considering seeking help in the second sample, as this is more of a result a not a core Methods aspect.

 Introduction: The aim of the study is not fully clear - is it to develop a diagnostic instrument to allow clinical assessment, or is it to measure burden of disease, and associated with that, what are the intended target populations? The approach, item response theory, is entangled with the content. I would suggest to first describe the aim and research gap more extensively, and then move towards the conceptual and empirical approach. A lot of the information provided here now seems more fitting for the Methods and Procedure sections.

 Methods section: I have no further comments on this section, besides moving some information from the Intro here (by adding a item construction section).

 Statistical Analysis: no further comments.

 Results: I would add more information on the two samples and how their socio-demographics overlap or differ in a table. Also it would help to know more about the sample with a much higher self-reported PH and their self-selection(?). Was sexual orientation assessed? If so, are there differences between heterosexual and LGBTQI+ samples? If not assessed, or underrepresented, the authors should add this to the limitation section that no sexual diversity was included. Given the unequal sample sizes, a sensitivity analysis could be conducted, or a random sample could be drawn to create equal sample sizes (and to be reported in the appendix)

 Discussion/Conclusion: The GD is well written and summarizes the results adequately. Here, the authors actually provide information on their aims in a much more succinct way than in the Introduction (from my perspective). Maybe it would help to include a para on the scope of aims, from diagnosis to a comprehensive construct to describe distress due to hypersexuality, in the Intro. At times, the DG and Conclusion read a bit more like a methods paper on IRT analyses. While critically assessing one's chosen method is fine, it should remain balanced toward the content. I like that the authors critically assess the problem of investigating a phenomenon with a low prevalence in the population. To gauge future relevance, maybe it would help to also make suggestions in how far changes in prevalence can be assessed, and how PH can be differentiated from popular (internet based) self-diagnoses.

If applicable, is the statistical analysis and its interpretation appropriate?

Partly

Is the study design appropriate and is the work technically sound?

Partly

Is the work clearly and accurately presented and does it engage with the current literature?

Partly

Are the conclusions drawn adequately supported by the results?

Yes

Are sufficient details of methods and analysis provided to allow replication by others?

Yes

Are all the source data and materials underlying the results available?

Partly

Reviewer Expertise:

sexual wellbeing, sexual health, self-regulation, substance use, sexual identities, discrimination

I confirm that I have read this submission and believe that I have an appropriate level of expertise to confirm that it is of an acceptable scientific standard, however I have significant reservations, as outlined above.

# Response

**Published:** 2024-06-05

**Authors:** Piet van Tuijl

**Affiliations:** Psychology, Open University, the Netherlands, Heerlen, Limburg, The Netherlands

To Prof. Kai Jonas, 17 ^th^ of June 2024

 Dear Kai Jonas,

 Thank you very much for your review! Your suggestions, we hope and think, will improve the work we have put forward significantly. Your suggestions mainly concerned clarification of the general aim of the study – and the lack of information on sexual orientation. We have tried to address both points and hope our adaptations can find your approval.

 The point where you mention the sensitivity analysis was not completely clear to us, as mentioned below.

**Comment reviewer** – The authors are presenting a MS proposing an improved way to measure problematic hypersexuality, using an item response theory approach. I would like the share the following comments with the authors:

 Abstract: I would remove the information about the percentage of those considering seeking help in the second sample, as this is more of a result a not a core Methods aspect.

**Reaction authors:**

 We do now not include this information anymore in the Methods section, but in the Results of the abstract.

**Comment reviewer** – Introduction: The aim of the study is not fully clear - is it to develop a diagnostic instrument to allow clinical assessment, or is it to measure burden of disease, and associated with that, what are the intended target populations? The approach, item response theory, is entangled with the content. I would suggest to first describe the aim and research gap more extensively, and then move towards the conceptual and empirical approach. A lot of the information provided here now seems more fitting for the Methods and Procedure sections.

**Reaction authors:**

 The aim is to develop to develop and validate a set of items mainly stemming from previously developed questionnaires for problems due to hypersexuality; the second aim aligns with this first aim – ideally: to arrive at a data-helped definition of PH. With the instrument that will thus be developed, the burden of PH can be measured as it uses the best – most cue valid – indicators for PH. The best set of items ideally should reflect the best indicators of PH – though there are caveats here, as has been mentioned in the discussion section with regard to withdrawal and tolerance.

 The two fold aim of the study has been added more clearly to the first paragraph of the introduction now:

*“* *We targeted an ideally aligning  twofold aim in this study: 1) psychometrically test items that measure problems due to hypersexuality – thus developing and validating item banks for PH; and 2) finetune the preliminary definition of PH, by taking into account previous conceptualizations – Sex Addiction, Hypersexual Disorder and Compulsive Sexual Behavior Disorder.”*

 A secondary aim of this study was to assess in what populations the questionnaire will work best. A questionnaire for PH should be able to assess prevalence of PH in the general population. However, importantly, it should first and foremost distinguish PH from other conditions in subpopulations that might closely resemble PH but are nonetheless significantly different from PH – like NH.

 In the paragraph concerning base rate of PH, we mention the discriminatory aim of the instrument and the intended target populations: those in doubt about their level of problems due to hypersexuality.

 We added the following text to the introduction with regard to the use of the instrument to measure the prevalence in the general population (to which we come back in the Discussion section). In the paragraph concerning the low base rate of PH in general population samples – the first challenge this study addresses – we added:

 “ *A secondary aim of such a discriminatory instrument can be to assess prevalence in the general population as well, though caution is advised when applying general population measurement to individual cases, e.g. as a screener.”*

 The research gap that we address concerns the conceptualization of PH and the lack of integration of previous conceptualizations of PH. Concerning “conceptualization”: we see PH as the convergence of criteria mentioned in three different perspectives on PH and we therefore included items that represent criteria from the three different perspectives on PH– this has been previously suggested but has not actually been done until now. Methodologically, the research gap we address regards the lack of use of relevant subpopulations and the lack of use of IRT to develop and validate questionnaires for PH. The methodological and conceptual research gaps are indeed entangled because they both led to design the current study.

 To clarify the research gap, we added:

*“* *The main research gap this study addresses concerns the lack of integration of different perspectives on PH. Empirical studies into the combined indicators of PH are lacking – hypersexuality studies mainly focused on one of the three perspectives mentioned above. The current study puts to practice what has been previously suggested (Montgomery-Graham, 2017) and combines different questionnaires previously developed for one of the three perspectives on PH. This combination allows us to test each questionnaires’ items in one study, not to pit the three perspectives against one another, but to converge – after extensive data analysis – toward an overarching concept that contains both the overlapping and unique indicators of each of the perspectives. Relating to the research gap mentioned above, there are also two methodological strategies we apply that have not been applied before to validate item banks for hypersexuality questionnaires (van Tuijl et al., 2023): 1) our study is the first to use IRT to develop and validate item banks to measure PH or related constructs; 2) we make use of the distinction between PH and Non-problematic Hypersexuality (NH; van Zessen, 1995; Štulhofer et al., 2016) to select the best items. Concerning IRT, we mention that the use of this technique allows for a continuous back-and-forth between data and construct as new items can be added anytime to the item banks. Concerning the distinction between PH and NH, this distinction allows us to select items that have the most discriminating power to discern these relevant groups from each other. Previous hypersexuality validation research did not use such group comparisons and thus could not unequivocally be expected to discern PH from NH. Both the use of IRT and the distinction between PH and NH will allow us to better address the main research gap – lack of integration of different perspectives on PH.”*

 The paragraph concerning IRT has been moved to the Method section, where it is to be read as a short methodological introduction to this method in the context of validation research. This is the paragraph that has been moved to the Method section:

*“Development and validation of scales using IRT.*

*In this study we use IRT to develop and validate items banks for PH, because this offers certain advantages over more common validation practices* *. IRT has previously been used to develop and validate scales for general health surveys questionnaires focused on constructs such as fatigue, pain, and depressive symptoms (Cella et al., 2007; Luijten et al., 2020) and similarly might be used to develop and validate an item bank for PH as well. In contrast to validation using only factor analysis, IRT allows for the ordering and selection of items based on item parameters (e.g. item difficulty or differentiating power). Because item difficulty and individuals’ latent trait scores are located on the same scale, it can be assessed which items are optimal to measure the trait in relevant target populations (e.g. Alons et al., 2022). Information gained from IRT can also be used to develop diverging short forms, serving discriminative or evaluative purposes (Terwee et al., 2007). No studies have been undertaken, to our knowledge, that use IRT to develop measurement instruments for PH or related constructs* *.”*

**Comment reviewer** – Methods section: I have no further comments on this section, besides moving some information from the Intro here (by adding a item construction section).

**Reaction authors:**

 The paragraph from the intro on item response theory has been moved here to the Methods section. Apart from 14 items we developed ourselves no items were constructed – maybe “scale construction section” is meant in the comment above? We moved the paragraph on IRT to the Method section and placed it under the heading “Development and validation of scales using IRT”.

 Furthermore, we added to the “Participants and procedure” paragraph of the Method section the following lines sexual orientation:

*”* *There are important differences in data collection between the two samples. In the general population sample the gender proportion was divided in women 48.9 %, men 51.0 %, other 0.1 %. In the web-based sample it was women 31.3 %, men 68.2 %, other 0.5 %. Furthermore, the percentages of Kinsey scale scores differed in both samples, with the general population sample showing more exclusively heterosexual participants than the web-based sample (82% versus 61%). In the supplemental material we have added a histogram to show the spread of Kinsey-scale scores in both samples and added more detailed information. While the general population sample can be taken to be representative of the Dutch population of 18 years and older, the web-based sample must be seen as a self-selected convenience sample. We can assume that all who have finished the questionnaire in the web-based sample,* *had a need for information and feedback pertaining to their problems due to hypersexuality.* *”*

**Comment reviewer **– Statistical Analysis: no further comments.

**Comment reviewer** – Results: I would add more information on the two samples and how their socio-demographics overlap or differ in a table. Also it would help to know more about the sample with a much higher self-reported PH and their self-selection(?). Was sexual orientation assessed? If so, are there differences between heterosexual and LGBTQI+ samples? If not assessed, or underrepresented, the authors should add this to the limitation section that no sexual diversity was included. Given the unequal sample sizes, a sensitivity analysis could be conducted, or a random sample could be drawn to create equal sample sizes (and to be reported in the appendix).

**Reaction authors:**

 In Table 1 most socio-demographics are presented for both samples. However, in that table we make a distinction between three groups and not between two samples. The second and third group (PH and NH) together make up the second sample (n=371 – web-based sample). This has been explained more clearly now. Also, differences between the two samples are mentioned now in the Results section.

 Sexual orientation was assessed with the Kinsey scale, and the averages for the three groups (GP/PH/NH) are reported in Table 1. The average of the Kinsey scale in the groups is presented there as well as the effect size of significant differences in Kinsey-scale scores in the three groups. The PH and NH groups together form the second sample. As mentioned before, we added to the supplemental material a histogram that illustrates the spread of Kinsey-scale scores in both samples – mentioned in the “Participants and procedure” paragraph of the Method section – and this shows for instance that the percentages of those identifying as exclusively heterosexual are higher in the general population sample than in the web-based sample. We added to this histogram also the percentages of women in each Kinsey scale category for each sample.

 We added to the current Differential Item Functioning analyses paragraph DIF analyses of sexual orientation. This shows that the items function similarly for participants that indicated to be exclusively heterosexual and those indicating they are not exclusively heterosexual and this result further validates the item banks for use in non-heterosexual populations. However, in order to perform these analyses, we had to dichotomize sexual orientation in “exclusively heterosexual” and “not exclusively heterosexual” – which is a limitation of the study and is mentioned in the limitations currently.

 We added to the supplemental material two interaction plots with sexual orientation on the x-axis and Emotion Dysregulation-PH or Negative Effects-PH on the y-axis – with the grouping variable (GP/PH/NH) as moderator. The interaction regression analyses pertaining to these plots are succinctly reported in the “Interpretability” paragraph of the Results section, and discussed in more detail in the supplemental material. The figures show that in the three groups an increase in Kinsey-scale score (1 = exclusively heterosexual – 7 = exclusively homosexual) goes together with an increase in Emotion Dysregulation-PH or Negative Effects-PH. In all groups there are more or less the same effects visible and the interaction is not significant for either ED-PH or NE-PH. Separate regression analyses per group and scale show that between 0.3 % and 3.9 % of variance in either Emotion Dysregulation-PH or Negative Effects-PH is explained by the Kinsey scale scores. This result is further discussed in the limitations of the Discussion section.

 In the limitation paragraph we added a further note on sexual orientation, expressing that the Kinsey scale might be rather limited in that bisexuality is seen as part of a conceptually continuous slider scale that goes from exclusively hetero- to exclusively homosexual. Thus, bisexuality is not considered as a separate sexual orientation. We also mention in the limitations the differences in percentages of women and men in the web-based sample, and differences in percentages of exclusively heterosexual and not exclusively heterosexual between the two samples.

 We are not entirely sure what should be addressed by the sensitivity analyses. They would not serve a purpose for the main part of the analyses, as far as we can see – item selection, CFA, IRT, interpretability, construct validity. We are open to further specification in this regard and await your suggestions.

**Comment reviewer** – Discussion/Conclusion: The GD is well written and summarizes the results adequately. Here, the authors actually provide information on their aims in a much more succinct way than in the Introduction (from my perspective). Maybe it would help to include a para on the scope of aims, from diagnosis to a comprehensive construct to describe distress due to hypersexuality, in the Intro. At times, the DG and Conclusion read a bit more like a methods paper on IRT analyses. While critically assessing one's chosen method is fine, it should remain balanced toward the content. I like that the authors critically assess the problem of investigating a phenomenon with a low prevalence in the population. To gauge future relevance, maybe it would help to also make suggestions in how far changes in prevalence can be assessed, and how PH can be differentiated from popular (internet based) self-diagnoses.

**Reaction authors:**

 We followed up on the advice to add a paragraph to the Introduction on the research gap that we address with this study. Immediately before that we added to the end of the first paragraph our research aims. We hope these are more clearly stated in the Introduction as well now.

 As our study concerns psychometric development and validation via IRT, mentioning IRT is hard to avoid in the Discussion section. Discussing psychometrics also aligns with our twofold research goals:

*“* *Our use of extensive samples of NH and PH participants further allowed us to pursue our twofold research goals: 1) psychometric development and validation of item banks for PH; and 2) update the preliminary definition of PH.”*

 Changes in prevalence might be assessed with the current instrument, but only at a group level – it should be avoided to draw conclusions about individual’s scores. As our research showed, despite selection of the best items/indicators that we could find, there is still a large overlap between PH and NH. This suggests that the item banks might be further improved. Nonetheless, the item banks we developed can already be used to assess prevalence. We noted in the Discussion that in particular responsiveness is important to investigate in future research. Such investigations will also show in how far change in prevalence can be attributed to momentary and coincidental fluctuations or can be attributed to higher or lower prevalence itself.

 The contentious issue of self-diagnoses such as “Porn addiction” or “Sex addiction” has been mentioned in the Discussion of the manuscript now. Taking sufficient caution, people in doubt about their level of problems due to hypersexuality can use the instrument to assess themselves:

*“* *All these results illustrate that questionnaire-based* *failsafe* *categorization of hypersexual individuals as problematic or not might not be justifiable at the moment. This is in particular important given there are numerous people who “diagnose” themselves as sex or pornography addicted and might experience negative consequences from this (Prause & Binnie, 2023). Therefore, the item banks we developed and validated should not be used for diagnosis, but should only be used as an instrument to preliminarily gauge the nature of someone’s hypersexual preoccupation. If used to self-assess, a doctor’s or sexologist’s view might be needed to complement the self-assessment.”*

 The Supplemental material  placed as extended data in the repository: https://osf.io/8x7vz/files/osfstorage

# Reviewer report: Reviewer response for version 1

**DOI:** 10.21956/openreseurope.17414.r35657

**Published:** 2023-11-15

**Authors:** Richard Krueger

**Affiliations:** 1 Columbia University, New York, New York, USA

This study reports on the use of item response theory to identify items that can be used to identify individuals with problematic hypersexuality and separate these from the general population and from non-problematic hypersexuality. 

 The methods are well described, appropriate informed consent obtained and results presented in a clear way. The literature cited is thorough, recent, and is cited for specific reasons.

 This is a valuable contribution given the current increase in research interest in hypersexual behaivor disorder.

 I am not that familiar with item response theory and with the various reasons items were selected and tested and I think that a statistician with expertise in this area should be consulted.

 From what I can see there are no corrections that I would suggest.

If applicable, is the statistical analysis and its interpretation appropriate?

I cannot comment. A qualified statistician is required.

Is the study design appropriate and is the work technically sound?

Yes

Is the work clearly and accurately presented and does it engage with the current literature?

Yes

Are the conclusions drawn adequately supported by the results?

Yes

Are sufficient details of methods and analysis provided to allow replication by others?

Yes

Are all the source data and materials underlying the results available?

Yes

Reviewer Expertise:

Paraphilic and compulsive sexual behavior disorders

I confirm that I have read this submission and believe that I have an appropriate level of expertise to confirm that it is of an acceptable scientific standard.
